# Regulatory miRNAs and lncRNAs in Skin Cancer: A Narrative Review

**DOI:** 10.3390/life13081696

**Published:** 2023-08-06

**Authors:** Nicole Natarelli, Aleena Boby, Shaliz Aflatooni, Jasmine Thuy Tran, Michael Joseph Diaz, Kamil Taneja, Mahtab Forouzandeh

**Affiliations:** 1 Morsani College of Medicine, University of South Florida, Tampa, FL 33602, USA; 2 School of Medicine, University of Indiana, Indianapolis, IN 46202, USA; jasmtran@iu.edu; 3 College of Medicine, University of Florida, Gainesville, FL 32610, USA; 4 Renaissance School of Medicine, Stony Brook University, Stony Brook, NY 11794, USA; 5 Department of Dermatology, University of Florida, Gainesville, FL 32606, USA

**Keywords:** non-coding RNA, lncRNA, microRNA, miRNA, skin cancer, melanoma

## Abstract

Non-coding RNAs (ncRNAs) have a significant regulatory role in the pathogenesis of skin cancer, despite the fact that protein-coding genes have generally been the focus of research efforts in the field. We comment on the actions of long non-coding RNAs (lncRNAs) and microRNAs (miRNAs) in the current review with an eye toward potential therapeutic treatments. LncRNAs are remarkably adaptable, acting as scaffolding, guides, or decoys to modify key signaling pathways (i.e., the Wnt/β-catenin pathway) and gene expression. As post-transcriptional gatekeepers, miRNAs control gene expression by attaching to messenger RNAs and causing their degradation or suppression during translation. Cell cycle regulation, cellular differentiation, and immunological responses are all affected by the dysregulation of miRNAs observed in skin cancer. NcRNAs also show promise as diagnostic biomarkers and prognostic indicators. Unraveling the complexity of the regulatory networks governed by ncRNAs in skin cancer offers unprecedented opportunities for groundbreaking targeted therapies, revolutionizing the landscape of dermatologic care.

## 1. Introduction

Cancer is the leading cause of death among United States adults aged 45–64 years [1]. Furthermore, cancer is the second leading cause of death among the following age groups: 1–14 years, 35–44 years, and 65+ years [1]. While various cancers are associated with high morbidity and mortality, research continues to contribute a greater understanding of cancer susceptibility, carcinogenesis, prognostic factors, and therapeutic strategies. In addition, recent studies have highlighted the significance of non-coding RNAs (ncRNAs) in biological functions and disease etiology.

**Figure 1:**
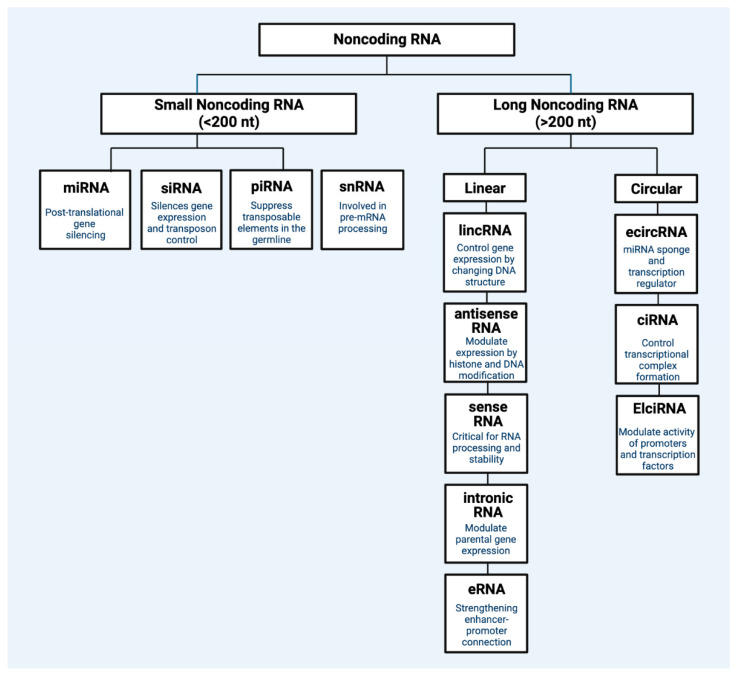
Classification of main regulatory non-coding RNA types. Regulatory ncRNAs are broadly divided into two general types: small non-coding RNA (<200 nucleotide) and long non-coding RNA (>200 nucleotides). Within each general type, there are different subtypes of non-coding RNA classes that each have specific mechanisms of gene control. miRNA: microRNA; siRNA: small interfering RNA; pirRNA: Piwi-interacting RNA; snRNA: small nuclear RNA; lincRNA: long intergenic non-coding RNA; eRNA: enhancer RNA; ciRNA: intronic RNA; ecircRNA: exonic circular RNA; ElciRNA: exon–intron circular RNA; Nt: nucleotide. Figure created with Biorender.com.

ncRNAs are transcriptome segments that do not code for proteins but perform regulatory roles in biological and disease processes [2]. Comprising about 98% of human transcriptional output [3], ncRNAs include long non-coding RNAs (lncRNAs), microRNAs (miRNAs), small interfering RNAs (siRNAs), piwi-interacting RNAs (piRNAs), promoter-associated transcripts (PATs), enhancer RNAs (eRNAs), and circular RNAs (circRNAs), among others [4]. ncRNAs can be classified by size, as lncRNAs have transcripts greater than 200 nucleotides, whereas small non-coding RNAs (sncRNAs) have transcripts of less than 200 nucleotides. ncRNAs can interact with other biomolecules, such as coding and non-coding RNAs, DNAs, and proteins, to exhibit influence on gene expression and biological processes. For example, while ncRNAs do not result in translated proteins, they can influence the expression of other protein-encoding genes [4]. Figure 1 captures the main regulatory ncRNAs.

### Non-Coding RNAs: miRNAs and lncRNAs

Of the various types of ncRNAs, a great proportion of research investigating their role in skin cancer details miRNAs and lncRNAs. MiRNAs are non-coding RNAs that function in gene expression regulation [5]. MRNA degradation and translational repression can be achieved via miRNA interaction with the 3′ untranslated region of target mRNAs. There have, however, been reports of miRNA interaction with other mRNA regions, such as the 5′ untranslated region, coding sequence, and gene promoters. In addition to degradation and repression, miRNAs can also activate translation or regulate transcription. MiRNAs located extracellularly may mediate cell–cell communication. Furthermore, they can be transported to target cells in vesicles or via protein-binding. Ultimately, the interaction of miRNA and targets is dependent on factors including the subcellular location and abundancy of miRNAs and the affinity of miRNA–mRNA interactions [5].

lncRNAs function similarly in gene regulation. As non-coding RNA, it is widely accepted that lncRNAs do not code for proteins in general, although it is necessary to note they can encode various micropeptides [6]. The regulating functions of lncRNA include the modulation of chromatin function, the regulation of the assembly and function of membraneless nuclear bodies, the alteration of stability and translation of cytoplasmic mRNAs, and interference with signaling pathways [7]. These functions may affect gene expression and influence the activity of neuronal disorders, immune responses, and cancer, providing further justification to investigate lncRNAs as biomarkers and target them clinically [7].

Skin cancer is the most common cancer globally. One in three diagnosed cancers is skin cancer. While keratinocyte carcinoma, such as basal cell carcinoma and squamous cell carcinoma, are more prevalent than melanoma, over 130,000 new cases of melanoma occur annually [8]. ncRNA expression in skin cancer, including miRNAs and lncRNAs, has gained significant attention due to its diagnostic and prognostic potential. Scientists have begun analyzing the differential expression of ncRNAs among skin cancer cell lines and investigating the functional roles of ncRNAs that are over- or under-expressed [9,10,11]. Identifying integral ncRNAs that promote carcinogenic processes, such as proliferation, invasion, or apoptosis inhibition, may foster the development of new therapeutic strategies. However, further research is needed to better understand the role of ncRNAs and how to translate novel findings into clinical practice.

As such, a sensitivity literature search of PubMed/MEDLINE was conducted on 6 April 2023. Medical Subject Heading (MeSH) and relevant keywords were searched using the Advanced Search Builder tool. The main search terms included “non-coding RNA” AND “skin cancer”. Additional articles were retrieved through the manual consultation of referenced papers. Priority was placed on highly cited and/or accessed articles written in English. Ultimately, this review seeks to describe discovered roles of ncRNA in skin cancer development and prognosis, including melanoma and non-melanoma skin cancer. We will describe the influence of ncRNA on disease susceptibility, cellular proliferation, invasion and metastasis, apoptosis, and prognosis. Lastly, we will discuss potential ncRNA-associated therapeutic strategies.

## 2. Melanoma

Melanoma is a tumor formed via the malignant transformation of melanocytes, the melanin-producing cells that reside at the basal layer of the epidermis. However, as melanocytes are derived from neural crest cells, non-cutaneous melanoma can arise in locations characterized via neural cell migration, such as the gastrointestinal tract and brain [12]. Melanoma in situ is associated with a 97% five-year survival rate, while stage IV melanoma has a 10% five-year survival rate. However, certain types of melanoma, such as acral lentiginous melanoma (ALM), are associated with increased mortality. ncRNAs have been implicated in various aspects of melanogenesis.

### 2.1. Biomarkers of Melanoma

LncRNAs regulate gene expression and may influence various cancer-promoting functions, such as proliferation, migration, and invasion. Their relative expression may therefore act as cancer biomarkers and provide diagnostic utility. For example, plasmacytoma variant translocation 1 (PVT1), an lncRNA that resides near the Myc oncogene, is differentially expressed in cutaneous melanoma [13]. In 2005, researchers analyzed 2 normal skin samples, 2 benign nevi, 2 atypical nevi, 2 melanoma in situ, and 8 melanoma and observed progressive increases in PVT1 levels from normal skin and benign nevi to atypical nevi, melanoma in situ, and malignant melanoma [14]. In addition, a 2017 study observed upregulated PVT1 in melanoma tissues compared to age- and gender-matched non-neoplastic nevi tissues [9]. Further analysis with 51 melanoma patients and 47 non-melanoma patients revealed the ability to discriminate patients from controls based on serum PVT1 levels with a sensitivity of 94.12%, depicting the potential diagnostic value of PVT1 in malignant melanoma.

In addition to PVT1, lncRNA small nucleolar RNA host gene 5 (SNHG5) has emerged as a potential melanoma marker. In 2015, researchers collected serum samples from 24 patients with malignant melanoma, 5 surgically treated patients who experienced recurrence, 15 healthy patients, and 5 patients with squamous cell carcinoma [10]. While the researchers used eight specific primers for lncRNAs, only serum levels of SNHG5 were significantly greater in melanoma patients compared to healthy controls (*p* < 0.001). Further analysis also revealed significantly greater SNHG5 levels in melanoma patients compared to patients with squamous cell carcinoma (*p* < 0.05). However, unlike the previously described PVT1 expression, serum SNHG5 levels did not increase based on melanoma staging. As such, the researchers concluded that the serum SNHG5 level acts as a biomarker for melanoma-bearing status, rather than a marker of cancer progression [10].

The upregulation of various other lncRNAs in melanoma have been described in the literature, such as Survival-Associated Mitochondrial-Melanoma-Specific Oncogenic Non-Coding RNA (SAMMSON), tyrosine-related protein 1 (TYRP1), Sprouty 4—intron 1 (SPRY4-IT1), urothelial-cancer-associated 1 (UCA1), metastasis-associated lung adenocarcinoma transcript 1 (MALAT-1), BRAF-activated non-coding RNA (BANCR), antisense non-coding RNA in the INK4 locus (ANRIL), HOX Transcript Antisense RNA (HOTAIR), and SRA-like non-coding RNA (SLNCR1) [11]. Similarly, a 2020 study performed a microarray analysis and found 4488 lncRNAs to be differentially expressed among acral lentiginous melanoma samples and adjacent non-tumor tissues (2211 upregulated; 2277 downregulated in ALM tissue) [15]. Such research depicts the potential utility of lncRNAs to act as melanoma markers and possible targets of therapeutic intervention. However, further research is necessary to determine the specificity and sensitivity of various markers when discriminating melanoma from non-melanoma.

### 2.2. Susceptibility

While the upregulation and downregulation of ncRNA may result from melanomagenesis itself, it is also possible that various ncRNA mutations confer susceptibility to the development of melanoma. A 2017 study used crowd-sourced data to identify melanoma-associated susceptibility loci. In total, 6628 cases and 287,591 controls were utilized for the analysis [16]. In addition to confirming 20/21 previously identified melanoma susceptibility loci at genome-wide significance, the authors discovered a single nucleotide polymorphism (SNP) rs187842643 within a non-coding RNA associated with melanoma (OR = 1.96; 95% CI = [1.54, 2.48]; p = 3.53 × 10^−8^). The ncRNA is located 177 kb downstream of brain-abundant-membrane-attached signal protein 1 (BASP1), and authors subsequently found reduced BASP1 expression in melanoma compared with benign nevi. Thus, this ncRNA may act as a susceptibility locus for malignant melanoma. Additional research is necessary to determine other ncRNAs’ susceptibility loci in melanoma.

### 2.3. Proliferation

Numerous studies have demonstrated the involvement of ncRNAs in the proliferation of melanoma cancer cells. In melanoma, elevated levels of specific ncRNAs have been identified in comparison to other normal tissues and cell lines, and this overexpression can lead to the increased proliferation of cancerous cells via various pathways. These findings emphasize the critical role of ncRNAs in melanoma development and progression. Targeting these ncRNAs may be a promising therapeutic strategy for melanoma treatment and an avenue for better diagnostic measures.

One such ncRNA is the BANCR, which was found to be highly expressed in melanoma tissues compared to melanocytic nevi and human melanocytes [17]. In this 2017 study, researchers discovered that microRNA-204 (miR-204), a suppressor of melanoma growth, is possibly a direct target of BANCR [17]. When BANCR was silenced in melanoma cells, levels of miR-204 were decreased [17]. Additionally, the study revealed that neurogenic locus notch homolog protein 2 (Notch2) is possibly a direct target of miR-204 and proposed the BANCR/miR-204/Notch2 axis as a mediator of cell proliferation and tumor progression [17].

Another ncRNA, PVT1 oncogene (PVT1), was also found to be upregulated in melanoma tissues compared to control [18]. However, silencing PVT1 inhibited cell proliferation and caused cell cycle arrest at the G0/G1 phase. In PVT1 knockdown cells, decreased levels of cyclin D1, N-cadherin, and vimentin were noted, while E-cadherin levels increased, indicating the significant role of PVT1 in cell proliferation [18]. The study also highlighted the possibility of PVT1 binding to enhancer of zeste homolog 2 (eZH2) in melanoma cells and regulating the expression of microrna-200 (mir-200c), proposing another pathway involved in cell proliferation [18].

Certain ncRNAs have been identified to interact with sex hormone receptors and differentially impact the progression, proliferation, and malignancy of melanoma cells based on gender [19]. One noteworthy lncRNA, SLNCR, has been observed to bind to the androgen receptor (AR), resulting in increased melanoma proliferation by modulating several growth regulatory genes [20]. This mechanism potentially accounts for the higher incidence of melanoma in men when compared to women [20]. Furthermore, the researchers suggest that this interaction might also explain the greater rates of melanoma metastases and lower overall survival rates observed in men [20]. Melanoma exhibits sex-related differences in incidence, prognosis, response to therapy, and overall survival [21], and emerging evidence suggests that ncRNAs regulated by sex hormones could contribute to these disparities.

### 2.4. Invasion and Metastasis

There is evidence that lncRNA plays an important role in promoting invasion and metastasis in melanoma. One such lncRNA is HOTAIR, which is highly expressed in lymph node metastatic melanoma tissues compared to primary melanoma tissues, with expression levels correlating with poor overall survival [22,23]. Tang et al. demonstrated that HOTAIR knockdown results in a reduction of motility and invasion in melanoma cells and causes a suppression of the degradation of the gelatin matrix [22].

Moreover, HOTAIR can target miRNAs to promote the metastasis of melanoma. HOTAIR competitively binds microRNA-152-3p (miR-152-3p), and results in the activation of the downstream PI3k/Akt/mTOR signaling pathway [23]. At the same time, expression levels of miR-152-3p were upregulated through the knockdown of HOTAIR and tumor metastasis of melanoma was inhibited, suggesting lncRNA HOTAIR can enhance metastasis through interference with specific miRNA [23].

LncRNAs have also been implicated in promoting epithelial–mesenchymal transition (EMT), allowing melanoma cells to migrate from the primary site. The silencing of HOTAIR led to increased expression of E-cadherin, an epithelial cell marker, and decreased expression of N-cadherin, a mesenchymal marker. Thus, HOTAIR can also promote melanoma cell invasion by regulating EMT [23]. Similarly, a separate study analyzing the knockdown of lncRNA H19 found a reversal of EMT in melanoma cell lines, suggesting H19 plays a role in promoting EMT and thus melanoma cell invasion [24].

### 2.5. Apoptosis

Multiple studies have assessed the effects of ncRNA expression on apoptosis in melanoma cells. A 2021 study evaluated the expression of lncRNA homeobox A11 antisense (HOXA11-AS) in cutaneous melanoma [25]. HOXA11-AS was upregulated in melanoma and was found to induce cell proliferation, metastasis, and epithelial–mesenchymal transition. In addition, apoptotic rate was determined via flow cytometry in A875 and M14 melanoma cells. While HOXA11-AS was associated with a low proportion of cells exhibiting apoptosis, apoptotic rate significantly increased upon HOXA11-AS knockdown (*p* < 0.05) [25].

Similarly, as growth-arrest-specific 6 antisense 2 (GAS6-AS2) lncRNA has been identified as a cancer-related lncRNA, researchers sought to identify the mechanisms of GAS6-AS2 in melanoma [26]. In addition to demonstrating increased GAS6-AS2 in melanoma cells, researchers found its expression to positively correlate with advanced stage and poor prognosis. Ectopic GAS6-AS2 expression inhibited apoptosis in melanoma cells, while GAS6-AS2 knockdown promoted apoptosis. Subsequent analysis found GAS6-AS2 to upregulate GAS6 expression and activate AXL/AKT/ERK signals. Confirming the role of GAS6 in mediating the effects of GAS6-AS2 lncRNA, GAS6-deficient cells reversed the ability of GAS6-AS2 overexpression to promote proliferation and inhibit apoptosis [26]. As such, researchers suggest that GAS6-AS2 may be a useful prognostic biomarker and therapeutic target in melanoma.

A 2011 study found SPRY4-IT1 lncRNA expression to be upregulated in melanoma cell lines compared to melanocytes and keratinocyte controls [27]. SPRY4-IT1 RNA knockdown affected cell growth and differentiation and increased apoptotic rates in melanoma cell lines, suggesting its role in the regulation of melanoma survival. Phosphatidylserine labeling with FITC-conjugated Annexin V was used to detect apoptosis. Increased siRNA transfection, resulting in SPRY4-IT1 knockdown, resulted in increased fractions of annexin-positive cells [27]. However, no significant differences were observed in propidium iodide-positive cells, suggesting cell death is primarily mediated via apoptosis, not necrosis.

Furthermore, a 2021 in vitro and in vivo study investigated the role of lncRNA TINCR ubiquitin domain containing (TINCR) in cutaneous melanoma [28]. The authors found significant downregulation of TINCR expression in melanoma, with advanced stages demonstrating greater downregulation. Melanoma cell lines A375 and MV3 were utilized for subsequent investigation as they demonstrated relatively lower TINCR expression compared to M14 cells. In addition to increased proliferation and invasion, TINCR underexpression, characteristic of melanoma cells, was associated with a significantly reduced percentage of apoptotic cells (*p* < 0.001). Further analysis found the regulation of large tumor suppressor kinase 1 (LATS1) expression via TINCR to mediate the observed effects. Small interfering RNAs were utilized to create a knockdown LATS1, siLATS1. While TINCR overexpression upregulated LATS1 mRNA expression levels, the presence of siLATS1 reversed these effects. In addition, co-transfection with siLATS1 in A375 cells and MV3 cells partially reversed the induction of the apoptosis characteristic of TINCR overexpression [28]. In other words, intact LATS1 is required for TINCR expression to effectively induce apoptosis. This study ultimately demonstrated reduced levels of lncRNA TINCR in melanoma, resulting in reduced apoptosis mediated by the regulation of LATS1 mRNA expression.

Lastly, in addition to lncRNA, microRNAs have been implicated in the regulation of apoptosis in melanoma cells [29]. In 2020, researchers found microRNA 143 (miR-143) expression to be downregulated in melanoma cancer cell lines WM115, SK-Mel-28, and A2058 compared to normal human epidermal melanocytes (*p* < 0.0001 for WM115 vs. control; *p* < 0.05 for SK-Mel-28 and A2058 vs. control). Subsequent experimentation with WM115 cell lines found that miR-143 transfection upregulated miR-143 and significantly reduced cell viability compared to control WM115, lacking miR-143 transfection (*p* < 0.01). The apoptosis rate was significantly increased in transfected cells (*p* < 0.0001) [29]. The results demonstrate the regulation of apoptosis, in addition to cell migration and metastasis, through miR-143 in melanoma cell lines. Collectively, these studies demonstrate the regulatory role of ncRNA in the apoptosis of melanoma cell lines.

### 2.6. Prognosis

ncRNAs can improve the prognosis prediction in melanoma. Several studies analyzed the prognostic value of lncRNA expression in melanoma patients through the development of prognostic prediction models. Immune-related, autophagy-related, and pyroptosis-related lncRNAs were selected and a prognostic risk model for melanoma was created to predict survival and prognosis. Using the risk model, patients were further classified into high-and low-risk groups according to the risk score [30]. The lncRNA risk signatures were found to be an independent prognostic factor with favorable prognostic ability for cutaneous melanoma [30,31,32,33,34,35,36,37].

Moreover, a recent study discovered that certain immune-related lncRNAs have been shown to have prognostic value in patients with melanoma. This study identified 7 immune-related lncRNAs (WAC-AS1, USP30-AS1, LINC01138, SPRY4-AS1, ZNF667-AS1, AC018553.1, and AC008060.3) that were differentially expressed in melanoma tissues compared to healthy skin tissues, and the authors also found that the expression of these lncRNAs was significantly associated with cancer prognosis [38]. Thus, measuring the expression of these immune-related lncRNAs could help predict the prognosis of melanoma patients and guide treatment decisions.

Other studies focused on the utility of specific lncRNAs as prognostic biomarkers. Huang et al. investigated the expression pattern and effects of lncRNA Down syndrome cell adhesion molecule antisense 1 (DSCAM-AS1) in melanoma and found that DSCAM-AS1 was significantly upregulated in melanoma tissues and cell lines [39]. Furthermore, DSCAM-AS1 was associated with an advanced stage and led to significantly poorer survival time [39]. The expression of ferroptosis-related lncRNA in melanoma tissues and cell lines was also explored. Risk assessment showed ferroptosis-related lncRNA is associated with poor prognosis [40]. Shi et al. investigated the role of lncRNA H19 in melanoma and found H19 was highly expressed in melanoma tissues compared to normal adjacent skin tissues and H19 expression levels were significantly higher in patients with metastatic melanoma compared to melanoma patients without distant metastasis (*p* < 0.01) [24]. Similarly, long intergenic ncRNA 173 (LINC00173) expression was significantly higher in melanoma tissues compared to adjacent non-neoplastic tissues (*p* < 0.01) and was expressed more frequently in melanoma patients with distant metastasis compared to patients at an earlier stage of melanoma [41]. Another study found that lncRNA LINC02249 expression was significantly elevated in cutaneous melanoma samples and a high expression of LINC02249 was associated with shorter overall survival in melanoma patients [42].

The quantification of miRNA expression can be useful In predicting prognosis in melanoma. Huang et al. and He et al. demonstrated poor survival in melanoma patients to be associated with the ability of lncRNA to reduce the expression of miRNA. Huang et al. showed that increased DSCAM-AS1 expression correlated with decreased microRNA-136 (mir-136) expression in melanoma tissue cells, and thus low expression of miR-136 was correlated with poor survival [39]. More recently, He et al. reported that microRNA-3662 (miR-3662) downregulates its target mRNAs and is associated with suppression of melanoma cell proliferation. LncRNAs negatively interact with miR-3662 and interfere with miR-3662 function, thus promoting proliferation [43].

Recent research has shed light on the role of several long non-coding RNAs (lncRNAs) in influencing tumor responses to cancer immunosurveillance and immunotherapy. These lncRNAs contribute to changes in the number, type, and activities of immune cell populations, impacting the effectiveness of immune responses against cancer [44]. Tumor cells often employ various mechanisms to evade host adaptive immunosurveillance, such as reducing the expression of major histocompatibility complex (MHC) class 1 antigens, increasing inhibitory checkpoint molecules, and minimizing the presentation of tumor-associated and tumor-specific antigens [45]. These same mechanisms can also contribute to cancer resistance to immunotherapy drugs [46]. For instance, a specific lncRNA called circ_0020710 has been found to correlate with cytotoxic lymphocyte exhaustion and resistance to anti-PD-1 therapy [47]. Likewise, increased expression of *MIR155HG* is directly linked to the heightened expression of immune checkpoint genes in melanoma, potentially compromising the effectiveness of melanoma immunotherapies [48]. These discoveries highlight the significance of ncRNAs in predicting responses to cancer therapy. Understanding these mechanisms may help improve the effectiveness of cancer treatments by enabling the prediction of patient responses to therapy.

## 3. Non-Melanoma Skin Cancer

### 3.1. Squamous Cell Carcinoma

Squamous cell carcinoma (SCC) is the second most common non-melanoma skin cancer. Local recurrence, regional metastases, distant metastases, and disease specific deaths occur at rates of 5, 5, 1, 1%, respectively [49]. Surgical excision is generally the standard treatment for low risk lesions. However, Mohs micrographic surgery is used for cSCC lesions on the head, neck, and other anatomically and cosmetically sensitive areas, and it has been reported to have better cure rates than a standard excision [50]. The 5-year survival rate for SCC is 99% when detected early.

**Figure 2:**
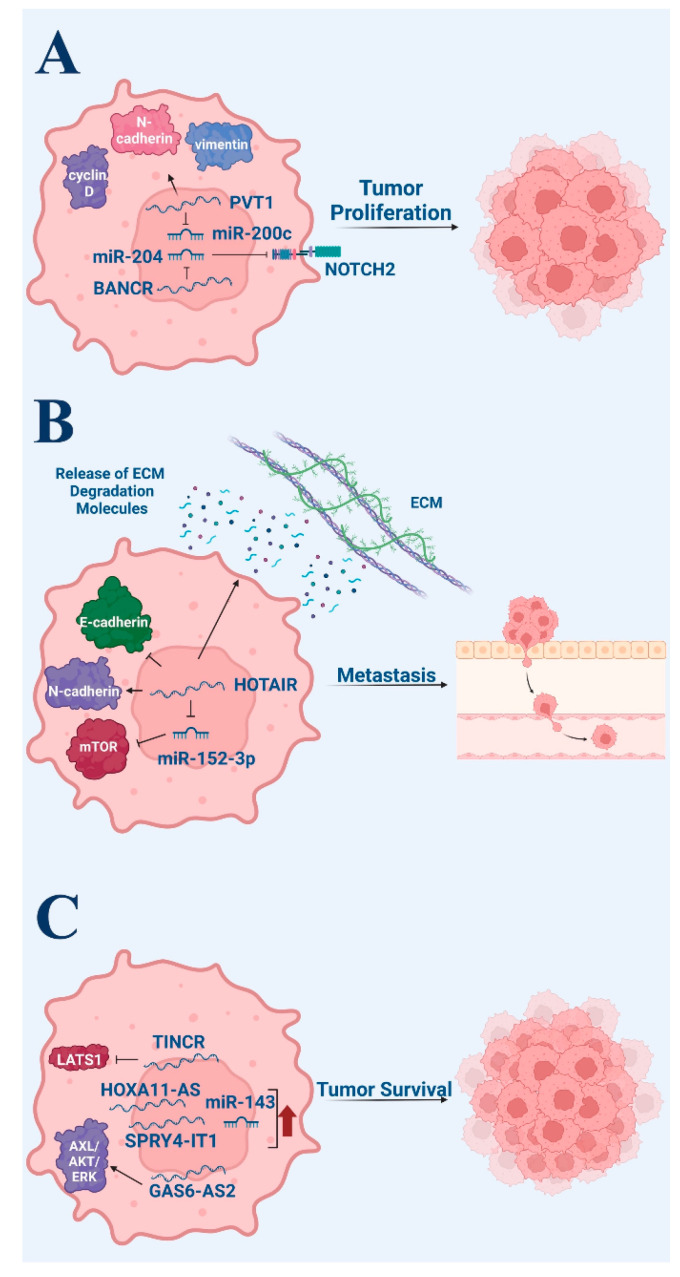
Pro-tumor roles of non-coding RNA in melanoma. (**A**) Tumor proliferation. (**B**) Tumor metastasis. (**C**) Tumor survival via anti-apoptotic signaling. Figure created with Biorender.com.

MiRNAs and lncRNAs are two types of ncRNAs that have both been explored in recent studies to discover the role it plays in the development and progression of non-melanoma skin cancer. MiRNAs have been identified as having both oncogenic and tumor suppressive roles (Figure 2), and dysregulation of these molecules has been implicated in the development of squamous cell carcinoma [51].

Studies have revealed the differential expression of lncRNAs in cancerous cells compared to normal cells. One study found 1516 upregulated and 2586 downregulated lncRNAs in cutaneous SCC cells compared to the control group [52], while another study by the same authors identified 1851 upregulated and 2165 downregulated lncRNAs in cSCC cells compared to controls [53]. The authors also found the increased expression of certain microRNA-17/92 (miR-17/92) members and tumor suppressor miR-143-5p in cSCC [54]. In a 2016 study, it was discovered that the expression of long intergenic ncRNA (LINC00162) was increased in tumor cells compared to keratinocytes in normal skin via the inhibition of the p38α and p38δ mitogen-activated protein kinases and appropriately named p38 inhibited cutaneous squamous-cell-carcinoma-associated lincRNA (PICSAR). The authors found that the knockdown of PICSAR inhibits the proliferation and migration of cSCC cells, highlighting the role it may play in cSCC development [55]. In a later study, the same authors discovered that the upregulation of PICSAR has been shown to promote the development of cSCC by decreasing the levels of certain integrins on the cell surface, resulting in increased cell proliferation [56].

Following this, a 2018 study revealed that the overexpression of miR-186 and decreased levels of apoptotic protease activating factor 1 (APAF1) are associated with the development of cSCC [57]. The overexpression of miR-186 was found to be associated with increased cell proliferation, migration, and invasion, while the knockdown of miR-186 was associated with decreased proliferation, migration, and invasion in cSCC cells [57]. Moreover, another study revealed a co-expression relationship between ACY3, NR1D1, MZB1, and six lncRNAs (GXYLT1P3, LINC00348, LOC101928131, A-33-p3340852, A-21-p0003442, and LOC644838) which could play a role in the progression of cutaneous squamous cell carcinoma [58]. These findings all highlight the importance of ncRNAs, such as miRNAs and lncRNAs, in the development and progression of cSCC, emphasizing the need to conduct further research in this field of study and unearth new diagnostic and prognostic avenues for non-melanoma skin cancer.

### 3.2. Basal Cell Carcinoma

Basal cell carcinoma is the most common human cancer worldwide. Basal cell carcinomas arise on sun-exposed skin, and while metastasis and fatality are rare, treatment and/or excision is required to prevent local destruction or disfigurement [59]. The 5-year survival rate is almost 100%, and fatal cases are often characterized by a lack of treatment or excision.

A 2021 study comparatively analyzed the differential expression of lncRNAs in basal cell carcinoma via whole-genome technology [60]. Of the 32,904 lncRNAs assessed, researchers found 1838 differentially expressed lncRNAs and confirmed the top 10 differentially expressed lncRNAs using reverse transcription-quantitative (RT-q) PCR. Subsequent Gene Ontology (GO) and Kyoto Encyclopedia of Genes and Genomes (KEGG) analysis found that lncRNA XR_428612.1 may function in mitochondrial dysfunction and the modulation of TICAM1, USMG5, COX7A2, FBXO10, ATP5E, and TIMM8B based on co-expression relationships [60]. The regulation of such genes may thereby promote basal cell carcinoma progression.

Similarly, a 2016 study retrieved punch biopsies from six patients with basal cell carcinoma to compare lncRNA expression among the tumor vs. NLES [61]. In total, 30,586 lncRNAs were screened, demonstrating a significant upregulation of 1851 lncRNAs and a significant downregulation of 2165 lncRNAs among basal cell carcinoma samples compared to non-lesional skin (*p* < 0.05) [61]. In total, 16 of 1205 assessed miRNAs were also found to be significantly upregulated in lesional skin among seven patients with basal cell carcinoma in a separate study [62]. Lastly, researchers identified 23 upregulated and 48 downregulated circRNAs after analysis of six basal cell carcinomas and six non-lesional healthy skin samples [52]. While additional research is required with increased sample size, the results of these studies confirm the dysregulation of lncRNAs, miRNAs, and circRNAs in basal cell carcinoma.

Additional studies have sought to uncover the functional role of ncRNAs that are differentially expressed in basal cell carcinoma. In 2018, authors observed significantly reduced microRNA 451a (miR-451a) in human basal cell carcinoma tissue and in a murine model [63]. miR-451a overexpression suppressed cell growth via G1 cell cycle arrest, and miR-451a inhibition fostered cellular growth and colony formation. Luciferase assay and protein expression analysis found T-box transcription factor 1 (TBX1) to be a downstream target of miR-451a, with levels inversely correlated with miR451a [63]. Another study found the upregulation of circular RNA Nck-associated protein 1 (circ_NCKAP1) in basal cell carcinoma tissues [64]. An in vitro study found the inhibition of cell proliferation and increased apoptosis with circ_NCKAP1 loss of function. Subsequent Dual-Luciferase reporter assay revealed direct binding capability between circ_NCKAP1 and microRNA-148b-5p (miR-148b-5p). Heat shock protein 90 (HSP90) is further targeted by miR-148b-5p, shedding light onto the potential mechanisms of circ_NCKAP1-associated proliferation and inhibition of apoptosis [64]. Lastly, the in vitro downregulation of microRNA-18a (miR-18a), a differentially elevated miRNA in basal cell carcinoma, was found to inhibit cell proliferation and activate autophagy through the Akt/mTOR signaling pathway. Conversely, miR-18a upregulation, characteristic of basal cell carcinoma, promoted cellular proliferation [65]. These studies demonstrate ncRNA dysregulation in basal cell carcinoma and highlight potential mechanisms in which ncRNA may regulate and promote carcinogenesis. Table 1 and Table 2 summarize studies detailing lncRNA and miRNA, respectively.

## 4. Potential Diagnostic and Therapeutic Strategies

**Figure 3:**
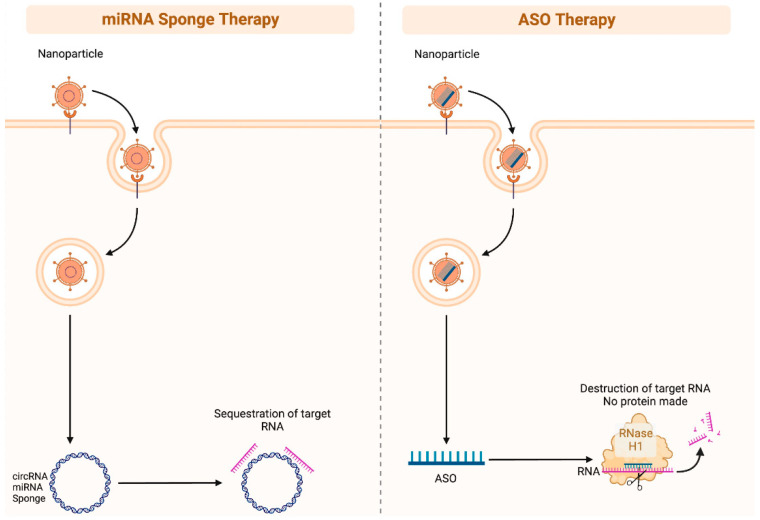
Illustration of miRNA sponge therapy and antisense oligonucleotides (ASO) therapy. miRNA sponges (often circular RNAs) can be administered as nanoparticles to patients, and they contain “sticky” regions that are designed to specifically bind to target RNAs. This is a potent method of silencing RNAs. ASOs are single strand DNA segments that are specifically designed to bind RNA. These DNA-RNA complexes can be recognized by the RNase H1 complex, which in turn will degrade the RNA. Figure created with Biorender.com.

As ncRNA biomarkers of skin cancer promote melanogenesis and carcinogenesis in various ways such as via the regulation of important proliferation, migration, invasion, and apoptotic pathways, such ncRNA may prove an effective therapeutic strategy. Therapeutic strategies may include ncRNA mimics and anti-ncRNAs. For example, miRNA inhibition can be achieved via miRNA sponges, antisense oligonucleotides, and small molecule inhibitors (Figure 3) [66]. However, effective and targeted delivery systems are necessary for ncRNA modulation specifically within cancer cells. Future research is necessary to assess the efficacy of therapeutic ncRNA targeting among different skin cancer cell lines in vivo and to characterize optimal targets and delivery systems.

Similarly, characteristic overexpression or underexpression of ncRNA may be useful to aid diagnosis or lesion differentiation. As previously described, researchers were able to effectively discriminate melanoma from non-melanoma based on PVT1 levels with a sensitivity of 94.12% [9]. In addition, a proof-of-concept study used miRNA fluorescence in situ hybridization (FISH) to differentiate between two tumor types: basal cell carcinoma and Merkel cell carcinoma [67]. Despite shared histologic features, basal cell carcinoma and Merkel cell carcinoma are of distinct cellular origins. The authors first assessed the differential miRNA expression between basal cell carcinoma, Merkel cell carcinoma, and normal skin. In addition to observing a 2-fold higher miRNA concentration in basal cell carcinoma, the authors identified basal-cell-carcinoma-specific microRNA-205 (miR-205) and Merkel-cell-carcinoma-specific miR-375. Using four basal cell carcinoma and 12 Merkel cell carcinoma cases, the authors performed a miRNA FISH with probes for 28S rRNA, miR-205, and microRNA-375 (miR-375). Using predetermined cutoff values for normalized fluorescence intensity, blinded analysis was able to correctly identify all evaluated cases [67]. This proof-of-concept study demonstrated the ability to use miRNA FISH techniques to differentiate between tumor tissues.

## 5. Conclusions

In the intricate realm of skin cancer, the dysregulation of ncRNAs emerges as a pivotal player. Diverse classes of ncRNAs, positioned as noteworthy regulators in the captivating landscape of skin cancer biology, take on the roles of either oncogenes or tumor suppressors, effectively modulating vital cellular processes that underlie the development of these diseases. Understanding the mechanism of ncRNA dysregulation may provide actionable insights into disease progression and uncover potential therapeutic targets. To fully appreciate the complex network of ncRNAs in skin cancer, additional investigation is yet needed. Increasing our understanding of these ncRNAs may realistically open the door to the development of novel diagnostic techniques and tailored treatments that will enhance the management and prognosis of skin cancer patients.

## Figures and Tables

**Table 1:** Summary of studies detailing lncRNA and skin cancer.

Cancer Type	Author (Year)	Study Type	N	Key Findings
Melanoma- biomarkers	Smith (2005) [14]	Genome expression comparative study	2 normal skin samples, 2 benign nevi, 2 atypical nevi, 2 melanomas in situ, 2 VPG melanomas, 2 MGP melanomas, 3 MGP-melanoma positive lymph nodes	PVT1 lncRNA differentially expressed in cutaneous melanomas
	Chen (2017) [9]	Genome expression comparative study via Gene Expression Oncomine database	51 melanoma patients and 47 non-melanoma patients	-Upregulated PVT1 in melanoma tissues vs. age- and gender-matched non-neoplastic nevi -PVT1 levels conferred the ability to discriminate patients from controls with 94.12% sensitivity
	Ichigozaki (2016) [10]	RNA expression comparative study	24 melanoma patients, 5 patients who experienced recurrence following surgical treatment, 15 healthy patients, and 5 patients with SCC	Significantly greater levels of lncRNA SNHG5 in melanoma patients compared to healthy controls (*p* < 0.001) and to patients with SCC (*p* < 0.001)
	Shi (2020) [15]	Genome expression comparative study	6 ALM patients: 1 tumor and 1 healthy adjacent tissue from each patient	4488 lncRNAs differentially expressed among ALM samples and adjacent non-tumor tissues (2211 upregulated; 2277 downregulated)
Melanoma- proliferation	Cai (2017) [17]	-RNA expression study -Murine study	69 advanced melanoma patients	-Low BANCR expression in melanocytic nevi and human melanocytes; high BANCR expression in melanoma -BANCR may promote cell growth through inhibition of miR-204, leading to Notch2 pathway activation
	Chen (2017) [18]	-RNA expression study -In vitro with melanoma cell lines A-375 and sk-mel-5	37 melanoma patients	-PVT1 highly expressed in melanoma tissues and cells -PVT1 silencing significantly decreased cyclin D1 and vimentin expression and significantly increased E-cadherin in melanoma cells -lncRNA PVT1 may contribute to tumorigenesis and metastasis of melanoma through binding to EzH2 and regulating the expression of miR-200c
Melanoma-invasion and Metastasis	Tang (2013) [22]	In vitro with melanoma cell line A375	N/A	HOTAIR knockdown results in reduced motility and invasion in melanoma cells and suppresses the degradation of the gelatin matrix
	Luan (2017) [23]	In vitro with melanoma cell line A375	N/A	HOTAIR promotes growth and metastasis of melanoma cells by competitively binding miR-152-3p
	Shi (2018) [24]	In vitro with melanoma cell lines A375 and 120Lu	N/A	Knockdown of lncRNA H19 resulted in reversal of EMT in melanoma cell lines
Melanoma-apoptosis	Xu (2021) [25]	-RNA expression comparative study -In vitro with melanoma cell lines A875 and M14	30 melanoma patients: 1 tumor and 1 healthy adjacent tissue from each patient	-HOXA11-AS (lncRNA) was upregulated in melanoma and was found to induce cell proliferation, metastasis, and EMT -Apoptotic rate significantly increased upon HOXA11-AS knockdown
	Wen (2019) [26]	-RNA expression comparative study -In vitro and murine study with melanoma cell lines A375, SK-MEL-2, and SK-MEL-5	56 melanoma tissues and 29 skin tissues with melanocytic nevus	-lncRNA GAS6-AS2 expression increased in melanoma cells and positively correlates with advanced stage and poor prognosis -Ectopic GAS6-AS2 expression inhibits apoptosis in melanoma cells -GAS6-AS2 knockdown promotes apoptosis
	Khaitan (2011) [27]	-RNA expression comparative study -In vitro with melanoma cell line WM1552C	29 graded melanoma patient samples and 6 normal skin samples	-Upregulated SPRY4-IT1 lncRNA in melanoma cell lines compared to melanocytes and keratinocyte controls -SPRY4-IT1 RNA knockdown increased apoptotic rates in melanoma cell lines -77 lncRNAs were significantly differentially expressed in WM1552C relative to melanocytes (*p* < 0.015)
	Han (2021) [28]	-RNA expression comparative study -In vitro with melanoma cell lines M14, A375, MV3	60 patients with cutaneous melanoma	-Significantly downregulated TINCR expression in melanoma, with advanced stages demonstrating greater downregulation -TINCR underexpression was associated with a significantly reduced percentage of apoptotic cells (*p* < 0.001) -TINCR regulation of LATS1 mediates observed effects
Melanoma-prognosis	Shi (2018) [24]	In vitro with melanoma cell lines A375 and 1205Lu	N/A	-lncRNA H19 was highly expressed in melanoma tissues compared to normal adjacent skin tissues -H19 expression levels were significantly higher in patients with metastatic melanoma compared to melanoma patients without distant metastasis (*p* < 0.01)
	Xue (2021) [30]	Genome expression study via The Cancer Genome Atlas database	446 cases	-HLA-DQB1-AS1, MIR205HG, RP11-643G5.6, USP30-AS1, and RP11-415F23.4 are independent prognostic factors of cutaneous melanoma -The low-risk group of the lncRNA signature showed a higher degree of immune infiltration, higher expression of immune checkpoint-associated genes, and better outcome of immunotherapy
	Qiu (2022) [31]	Genome expression study via The Cancer Genome Atlas database	470 cases	-Risk model developed using 15 autophagy-related lncRNAs (LINC01943, AC090948.3, USP30-AS1, AC068282.1, AC004687.1, AL133371.2, AC242842.1, PCED1B-AS1, HLA-DQB1-AS1, AC011374.2, LINC00324, AC018553.1, LINC00520, DBH-AS1, and ITGB2-AS1) -Negative correlations between risk scores and overall survival rate in melanoma patients
	Chen (2017) [32]	Genome expression study via The Cancer Genome Atlas database and the GSE65904 database	458 cases (Cancer Genome Atlas) and 210 cases (GSE65904)	-The four-lncRNA (HCP5, LIMD1-AS1, *MIR155HG*, and UNQ6494) can be used as prognostic factors for cutaneous melanoma -The duration of survival in patients in high-risk groups were significantly shorter than that of low-risk groups
	Wu (2021) [33]	Genome expression study via The Cancer Genome Atlas database	470 cases	-A risk signature based on 22 pyroptosis-related lncRNAs was generated -The risk signature was significantly correlated with immune microenvironment and immune cell infiltration
	Xiao (2021) [34]	Genome expression comparative study via The Cancer Genome Atlas database	471 cases and 1 control	-Eight immune-related lncRNAs were used to build a prognostic risk signature model; AC091729.3, AC245595.1, LINC02560, and PCED1B-AS1 were risk-associated, whereas AC242842.1, AL133371.2, HLA-DQB1-AS1, and LINC01871 were protective -High-risk group showed lower levels of CD8+ T cells, M1 macrophages, plasma cells, and mast cells
	Yang (2019) [35]	Genome expression study via The Cancer Genome Atlas database	470 cases	-lncRNA MIAT was overexpressed in melanoma and promoted cell proliferation, cell invasion, and migration -The knockdown of MIAT expression decreased cell proliferation, invasion, and migration
	Zhang (2020) [36]	Genome expression comparative study via The Cancer Genome Atlas and Genotype Tissue Expression databases	471 cases and 813 controls	-Found more than 10 lncRNA statistically significantly associated with patient survival rate -Using area under the curve, authors found lncRNAs FOXD2-AS1, MALAT1, NEAT1, AC245595.1, and SNHG5 have the most significant prognostic value
	Zi (2023) [37]	Genome expression study via The Cancer Genome Atlas database	459 cases	Patients with high risk scores predicted by lncRNA signatures tend to have high BHLHE40-AS1 expression and low RP11-677M14.7, RP11-326I11.3, RP1-151F17.2, RP11-1094M14.5, HCP5, FUT8-AS1, and RP11-6O2.3 expression
	Guo (2021) [38]	Genome expression comparative study via The Cancer Genome Atlas database	Not stated	-Expression levels of ZNF667-AS1, AC008060.3, and AC018553.1 were gradually increased with increasing risk scores, while expression levels of WAC-AS1, USP30-AS1, LINC01138, and SPRY4-AS1 were gradually reduced -Survival curve analysis showed that the survival time of patients was significantly shorter in the ZNF667-AS1, AC008060.3, and AC018553.1 high expression group than the low expression group
	Huang (2019) [39]	RNA expression comparative study with melanoma cell lines 1205Lu, CHL-1, A-375, UACC903, and SK-MEL-2 and normal human epidermal melanocyte HEMa-LP	N/A	-lncRNA DSCAM-AS1 expression was associated with ulceration and advanced stage and led to significantly poorer survival time -High DSCAM-AS1 expression in melanoma is an independent predictor of poor survival of patients -Increased DSCAM-AS1 expression correlated with decreased mir-136 expression in melanoma tissue cells, and thus low expression of miR-136 was correlated with poor survival
	Rao (2022) [40]	Genome expression study via The Cancer Genome Atlas database	472 cases and 810 controls	-Ten ferroptosis-related lncRNA risk models were significant prognostic factors for patients with melanoma and predive factors for overall survival -LINC00520 is an adverse prognostic factor for melanoma. Survival analysis showed that LINC00520 is highly expressed in melanoma in the high risk group -ROC curve suggested the risk score has reliable predictive ability (AUC = 0.718)
	Wang (2022) [41]	-In vitro with paired cancerous and non-cancerous specimen samples	163	-LINC00172 was highly expressed in melanoma specimens compared to adjacent non-neoplasm specimens -LINC00172 was highly expressed in patients with advanced melanoma compared to patients with early melanoma -LINC00172 expression level is an independent prognostic predictor of melanoma
	Du (2022) [42]	Genome expression comparative study via The Cancer Genome Atlas database	471 cases and 1 control	-LINC02249 was highly expressed in cancerous specimens compared to non-cancerous specimens -High expression of LINC02249 was associated with shorter overall survival and disease-specific survival of melanoma patients -LINC02249 was an independent prognostic factor for melanoma -Expression of LINC02249 was negatively associated with tumor-infiltrating immune cells
	He (2021) [43]	In vitro with melanoma cell line A375	N/A	-lncRNAs negatively interact with miR-3662 and interfere with miR-3662 function, thus promoting proliferation
Squamous Cell Carcinoma	Sand (2016) [53]	RNA expression comparative study	6 patients with SCC and adjacent healthy skin samples	A total of 1516 upregulated lncRNAs and 2586 downregulated lncRNAs in cSCC compared with controls
	Piipponen (2016) [55]	RNA expression study	8 cSCC and 4 normal human epidermal keratinocytes	-cSCC LINC00162 expression upregulated via the inhibition of the p38α and p38δ mitogen-activated protein kinases -LINC00162 expressed by tumor cells in cSCCs but not by keratinocytes in normal skin -Knockdown of LINC00162 inhibited proliferation and migration of cSCC cells -Knockdown of LINC00162 inhibited extracellular signal-regulated kinase 1/2 activity and upregulated expression of DUSP6 in cSCC cells - Proposed role of PICSAR in promoting cSCC progression via the activation of extracellular signal-regulated kinase 1/2 signaling pathway by downregulating DUSP6 expression
	Piipponen (2018) [56]	RNA expression comparative study	Not specified	- Knockdown of PICSAR in cSCC cells upregulates expression of α2, α5, and β1 integrins; downregulation with overexpression of PICSAR -Downregulation leads to decreased cell adhesion on collagen I and fibronectin and increased cell migration
	Hu (2022) [58]	RNA expression study	6 patients with cSCC	-mRNAs ACY3, NR1D1, and MZB1 have a co-expression relationship with six lncRNAs, GXYLT1P3, LINC00348, LOC101928131, A-33-p3340852, A-21-p0003442, and LOC644838
Basal Cell Carcinoma	Zhang (2021) [60]	Whole genome expression comparative study	13 patients with BCC and 13 healthy volunteers	In total, 1838 (of 32,904 lncRNAs assessed) were differentially expressed
	Sand (2016) [61]	RNA expression comparative study	6 patients with BCC and adjacent healthy skin samples	In total, 1851 lncRNAs significantly upregulated and 2165 lncRNAs significantly downregulated among BCC samples vs. non-lesional skin (*p* < 0.05) (30,586 lncRNAs assessed)
	Sand (2016) [52]	RNA expression comparative study	6 patients with BCC and adjacent healthy skin samples	A total of 23 upregulated and 48 downregulated circRNAs
	Fan (2021) [64]	RNA expression comparative study	Three pairs of skin BCC tissues and adjacent tissues	-Upregulated circ NCKAP1 in BCC tissues -Inhibition of cell proliferation and increased apoptosis with circ_NCKAP1 loss of function -Direct binding capability between circ_NCKAP1 and miR-148b-5p -HSP90 is targeted by miR-148b-5p

Alm: acral lentiginous melanoma; auc: area under the curve; bcc: basal cell carcinoma; circrna: circular rna; cscc: cutaneous squamous cell carcinoma; emt: epithelial-mesenchymal transition; lncrna: long non-coding rna; mrna: messenger rna; roc: receiving operating characteristic; scc: squamous cell carcinoma; snp: single nucleotide polymorphism; vpg: vertical growth phase.

**Table 2:** Summary of studies detailing miRNA and skin cancer.

**Cancer Type**	**Author (Year)**	**Study Type**	**N**	**Key Findings**
Melanoma	Cai (2017) [17]	-RNA expression study -Murine study	69 advanced melanoma patients	-miR-204 (suppressor of melanoma growth) downregulation in melanoma tissues and cell lines -BANCR may promote cell growth through the inhibition of miR-204, leading to Notch2 pathway activation
	Nabipoorashrafi (2020) [29]	-RNA expression comparative study -In vitro with melanoma cell lines WM115, NK-Mel-28, and A2058 vs. normal skin	N/A	-Downregulated microRNA miR-143 expression in melanoma cell lines compared to NHEM normal skin -miR-143 transfection upregulated miR-143 and significantly reduced cell viability compared to control WM115 (*p* < 0.01) -Apoptosis significantly increased in transfected cells (*p* < 0001)
	He (2021) [43]	In vitro with melanoma cell line A375	N/A	-miR-3662 downregulates its target mRNAs and is associated with suppression of melanoma cell proliferation. -lncRNAs negatively interact with miR-3662 and interfere with miR-3662 function, thus promoting proliferation
Squamous Cell Carcinoma	Sand (2012) [62]	RNA expression comparative study	15 patients with cSCC and 16 control specimens from non-lesional skin	Significantly increased expression of miR-17-92 members miR-17-5p, miR-18a-5p, miR19a-3p, and miR-19b-3p and tumor suppressor miR-143-5p in cSCC (*p* < 0.01)
	Tian (2018) [57]	RNA expression comparative study	15 paired tumor and adjacent normal tissues	- MiR-186 expression significantly increased, while APAF1 expression significantly decreased in cSCC tissues compared with the controls. - Cell proliferation, invasion, and migration significantly increased in the miR-186-overexpressed A-431 cells and decreased in miR-186 knockdown cells compared to control - APAF1 expression regulated by miR-186, while APAF1 knockdown significantly promoted cell invasion and inhibited cell apoptosis - miR-186 serves as oncogene in cSCC by inhibiting APAF1
Basal cell carcinoma	Sand (2012) [62]	miRNA expression comparative study	7 patients with BCC and adjacent healthy skin samples	In total, 16 of 1205 assessed miRNAs significantly upregulated in lesional skin
	Sun (2018) [63]	-RNA expression study -Murine study	22 BCC patients	-Significantly reduced miRNA-451a in human BCC tissue and in a murine model -miRNA-451a overexpression suppressed cell growth via G1 cell cycle arrest, and miRNA-451a inhibition fostered cellular growth and colony formation -Luciferase assay and protein expression analysis found TBX1 to be a downstream target of miRNA-451a, with levels inversely correlated with miR451a.
	Mi (2020) [65]	-RNA expression comparative study via Gene Expression Omnibus database -In vitro with cell line A431	-7 BCC and 7 controls from Omnibus -Tissue collected from 20 patients with BCC and 20 healthy controls	Downregulation of miRNA iR-18a (differentially elevated in BCC) was found to inhibit cell proliferation and activate autophagy through the Akt/mTOR signaling pathway -miR-18a upregulation, characteristic of BCC, promoted cellular proliferation

Bcc: basal cell carcinoma; cscc: cutaneous squamous cell carcinoma; lncrna: long non-coding rna; mirna: micro rna; mrna: messenger rna; scc: squamous cell carcinoma; snp: single nucleotide polymorphism.

## Data Availability

Data sharing is not applicable to this article.

## Abbreviations

ALM	Acral lentiginous melanoma
ANRIL	antisense non-coding RNA in the *INK4* locus
BANCR	BRAF-activated non-coding RNA
BASP1	Brain abundant membrane attached signal protein 1
circRNA	circular RNA
Circ_NCKAP1	circular RNA Nck-associated protein 1
DSCAM-AS1	Down syndrome cell adhesion molecule antisense 1
EMIT	epithelial-mesenchymal transition
eRNA	enhancer RNA
eZH2	enhancer of zeste homolog 2
FISH	luorescence in situ hybridization
GAS6-AS2	growth arrest specific 6 antisense 2
GO	Gene ontology
HOTAIR	HOX Transcript Antisense RNA
HOXA11-AS	Homeobox A11 antisense
HSP90	heat shock protein 90
KEGG	Kyoto Encyclopedia of Genes and Genomes
LATS1	Large tumor suppressor kinase 1
LINC00173	Long intergenic non-coding RNA 173
lncRNA	long non-coding RNA
MALAT-1	metastasis associated lung adenocarcinoma transcript 1
microRNA	miRNA
miR-18a	microRNA-18a
miR-136	microRNA-136
miR-143	microRNA-143
miR-148b-5p	microRNA-148b-5p
miR-152-3p	microRNA-152-3p
miR-451a	microRNA-451amiR-204 = microRNA-204
miR-200c	microRNA-200
miR-3662	microRNA-3662
miR-17/92	microRNA-17/92
miR-205	microRNA-205
miR-375	microRNA-375
ncRNAs	non-coding RNAs
Notch2	neurogenic locus notch homolog protein 2
PAT	promoter-associated transcripts
piRNA	piwi-interacting RNA
PVT1	Plasmacytoma variant translocation 1
RT-q	Reverse transcription-quantitative
SAMMSON	Survival Associated Mitochondrial Melanoma Specific Oncogenic
SCC	squamous cell carcinoma
SNHG5	small nucleolar RNA host gene 5
TBX1	T-box transcription factor 1
siRNA	Small interfering RNA
SLNCR1	SRA-like non-coding RNA
sncRNA	small non-coding RNA
TINCR	TINCR ubiquitin domain containing
TYRP1	tyrosine related protein 1
UCA1	urothelial cancer associated 1
